# The Short-Term Efficacy of a Three-Week Pulmonary Rehabilitation Program among Patients with Obstructive Lung Diseases

**DOI:** 10.3390/jcm13092576

**Published:** 2024-04-27

**Authors:** Magdalena K. Klimczak, Hubert A. Krzepkowski, Wojciech J. Piotrowski, Adam J. Białas

**Affiliations:** 1 Department of Pneumology, Medical University of Lodz, 90-419 Lodz, Poland; magdalena.klimczak@umed.lodz.pl (M.K.K.); krzepkowskihubert@gmail.com (H.A.K.); wojciech.piotrowski@umed.lodz.pl (W.J.P.); 2 Department of Pulmonary Rehabilitation, Regional Medical Center for Lung Diseases and Rehabilitation, Blessed Rafal Chylinski Memorial Hospital for Lung Diseases, 91-520 Lodz, Poland

**Keywords:** pulmonary rehabilitation, PR, obstructive lung disease, asthma, chronic obstructive pulmonary disease, COPD, the coexistence of asthma and COPD

## Abstract

**Introduction**: The recommended duration for pulmonary rehabilitation stands at a minimum of six weeks; however, this stipulation may pose constraints in various countries due to financial limitations imposed by insurance companies and/or national health funds, as is the case in Poland. Consequently, our study endeavors to analyze the short-term outcomes stemming from a condensed three-week PR regimen administered to patients diagnosed with chronic obstructive pulmonary disease (COPD), asthma, and the concomitance of these conditions (COPD-A)—this is an approach that is standard in the rehabilitation protocols endorsed by our national health fund. **Methods**: Patients diagnosed with COPD, asthma, and COPD-A, referred to the PR program, underwent retrospective analysis to evaluate the short-term efficacy of a three-week PR program. Patients underwent comprehensive assessment by respiratory physicians and rehabilitation consultants, leading to individualized PR programs. Clinical evaluations occurred at program onset and completion. **Results**: 125 patients participated: 37 COPD, 61 asthma, and 27 COPD-A. Significant improvements were observed in the COPD Assessment Test (CAT), the consensus-based GINA symptom control tool (GINA-SCT), the Modified Medical Research Council (mMRC) scale, forced expiratory volume in the first second (FEV1), forced vital capacity (FVC), and the 6-min walk test (6 MWT) distance, as well as in the St. George’s Respiratory Questionnaire (SGRQ) scores. All groups experienced reduced dyspnea severity and improved exercise tolerance. FEV1 and FVC improved in asthma and COPD-A, but not significantly in COPD. Multivariable logistic regression identified predictive factors for PR response. **Conclusions**: The study supports the short-term efficacy of the three-week PR program in improving clinical outcomes, exercise tolerance, and quality of life in COPD and asthma patients. Tailoring interventions based on predictors of PR response can optimize outcomes. Further research, particularly of the COPD-A group, is needed for individualized approaches. Larger sample sizes are necessary to confirm our findings.

## 1. Introduction

The treatment of chronic obstructive lung diseases represents a paramount concern within the realm of respiratory care on a global scale. According to the current definition, Chronic Obstructive Pulmonary disease (COPD) manifests as a heterogeneous pulmonary disorder typified by persistent respiratory symptoms stemming from aberrations in the airways and/or alveoli, thereby inducing enduring and frequently progressive airflow obstruction [1]. Management of this disease encompasses a multifaceted approach, integrating pharmacotherapy and lifestyle interventions, including smoking cessation and structured rehabilitation regimens [1,2]. Pharmacological interventions predominantly focus on bronchodilation via beta-agonists and anticholinergics, supplemented by inhaled corticosteroids (ICS) in specific patient subsets [3].

Conversely, asthma constitutes another heterogeneous condition, commonly characterized by chronic airway inflammation. It is delineated by a history of respiratory manifestations, including wheezing, dyspnea, chest tightness, and coughing, which exhibit variability over time and in intensity, alongside variable expiratory airflow limitation, which may transition to a persistent state [4]. The cornerstone of pharmacological intervention resides in the application of ICS-formoterol as a maintenance and reliever therapy [4,5]. In cases of severe asthma, combinations involving high-dose ICS,d long-acting beta2 agonists, and add-on long-acting muscarinic antagonists or azithromycin, may be considered. Additionally, individualized assessments may justify the utilization of biological treatments, such as omalizumab, mepolizumab, benralizumab, or tezepelumab [4,6].

Moreover, asthma is acknowledged as a predisposing factor for the onset of COPD, as underscored by the identification of a distinct ethiotype, namely COPD-A, in accordance with the guidelines outlined by the Global Initiative for Chronic Obstructive Lung Disease (GOLD). *Ipso facto*, asthma and COPD may coexist in an individual patient [1]. 

A plethora of evidence exists regarding the efficacy of Pulmonary Rehabilitation (PR) in patients afflicted with asthma and COPD [7,8,9,10,11,12,13,14,15]. Nonetheless, rigorous evaluation of PR in the context of concomitant manifestation of these two diseases has been lacking, prompting a call for further investigation within the literature [16].

The recommended duration for pulmonary rehabilitation stands at a minimum of six weeks; however, this stipulation may pose constraints in various countries, due to financial limitations imposed by insurance companies and/or national health funds [1], as is the case in Poland. Consequently, our study endeavors to analyze the outcomes stemming from a condensed three-week PR regimen administered to patients diagnosed with COPD, asthma, and the concomitance of these conditions (COPD-A)—an approach standard in the rehabilitation protocols endorsed by our national health fund.

## 2. Materials and Methods

Patients diagnosed with COPD, asthma, and COPD-A, referred to the Department of Pulmonary Rehabilitation at Blessed Rafal Chylinski Memorial Hospital for Lung Diseases in Lodz, Poland, underwent retrospective analysis to evaluate the effectiveness of a three-week PR program. Diagnosis of asthma, COPD, or their coexistence was established following recommendations outlined by the Global Initiative for Asthma (GINA) or the Global Initiative for Chronic Obstructive Lung Disease (GOLD) reports. In the case of COPD-A, patients were reevaluated to ensure concordance with the current statement on this issue [1,4].

Each patient underwent a thorough assessment, conducted by an experienced respiratory medicine physician and a medical rehabilitation consultant, leading to the development of an individualized, multidimensional PR program tailored to specific needs. The PR regimen comprised of a 15-min warm-up, followed by interval endurance training on a cycle ergometer and rotors for the upper and lower limbs, lasting 30 min, which was conducted five days a week. The training intensity was set at 55–74% of maximum heart rate, incorporating intervals structured with a 1:2 work-rest ratio. Endurance training sessions were complemented with relaxation and calming exercises lasting 15 min. 

Resistance training, utilizing resistance bands for a total body workout, was conducted three days a week. Initially, the resistance was set at 40% of one-repetition maximum, with 4 sets of 5–8 repetitions and 2–3 min of rest between sets. Additionally, supervised exercises targeting the activation and strengthening of inspiratory and expiratory muscles, as well as the diaphragm, were performed. Each resistance training session was followed by stretching exercises lasting 10–30 s for each exercise, with 2–4 repetitions. Flexibility exercises, balance, and coordination training were administered through bi-weekly circuit training sessions. Generally, this part of training lasts about 45–60 min. Training progression was individually adjusted according to recommendations by the European Respiratory Society (ERS) and the American Thoracic Society (ATS) [17].

Furthermore, the program encompassed reeducation on breathing patterns, instruction on proper cough techniques, utilization of vibration pillows, and positional drainage, followed by chest tapping post-nebulization with a mucolytic agent. 

Patients requiring psychological support were referred to a clinical psychologist for consultation. Moreover, all patients underwent routine assessments of nutritional status and received guidance from an experienced clinical dietitian, with dietary adjustments tailored to individual requirements and overall health condition. Each patient also received education on correct inhaler usage and gained comprehensive knowledge about their respective lung diseases. This comprehensive approach aimed to address various aspects of patient well-being, including physical training, respiratory education, psychological support, nutritional guidance, and disease management education. Additionally, all patients received a tailored home exercise recommendations aimed at facilitating their transition from supervised physical activity to an autonomous, home-based program, thus ensuring the sustained accrual of therapeutic benefits following program completion.

Patients underwent two-point clinical assessments: one at the onset and another upon completion of the three-week rehabilitation program, with the initial examination encompassing diagnostic confirmation. Pulmonary assessment primarily involved spirometry with a reversibility test, utilizing reference values from the Global Lung Function Initiative (GLI-2012) [18]. Whole-body plethysmography was conducted in cases of suspected mixed restrictive-obstructive abnormalities. 

Patients diagnosed with COPD underwent evaluation using the COPD Assessment Test (CAT), while those diagnosed with asthma were subject to assessment employing the consensus-based GINA symptom control tool (GINA-SCT). For individuals presenting with the coexistence of asthma and COPD, both assessments were utilized. Exercise tolerance was evaluated through a 6-min walk test (6 MWT), with an improvement of 45 m or more considered significant [19,20], conducted in a standardized manner [21]. Dyspnea was quantified using the Modified Medical Research Council (mMRC) scale [22], while health-related quality of life was assessed using the St. George’s Respiratory Questionnaire (SGRQ) [23]. 

Only patients completing the PR program were included in the analysis. 

Statistical analysis was performed using R software ver. 4.2.3 [24] for macOS. Continuous data were presented as means with standard deviations (SDs) or medians with interquartile ranges [IQRs], contingent upon data distribution. Between-group comparisons utilized unpaired Student’s t-test, Welch t-test, Wilcoxon rank-sum test, Kruskall–Wallis test, or ANOVA, depending on data normality and variance homogeneity. Paired data were assessed using paired t-test or Wilcoxon signed rank test. Categorical data were analyzed using Pearson’s Chi-squared test or Fisher’s Exact Test. Missing data were not imputed in the analysis. Logistic regression analysis was employed to evaluate study parameters in predicting significant improvement in 6 MWT distance defined as 45 m), with forward and backward stepwise selection approaches used to refine the model. Receiver operating characteristics (ROC) and area under the ROC curve (AUROC) analyses were conducted to determine the predictive power of the regression model.

Corrections for multiple comparisons were not used due to the pre-defined hypotheses and comparisons that were specified before data collection. Missing data were not imputed.

The study protocol received approval from the Bioethical Committee of the Medical University of Lodz (RNN/257/21/KE), with patient consent waived due to the purely retrospective nature of the study.

## 3. Results

### 3.1. Baseline Data Analysis

In total, 125 patients were enrolled in the study: 37 (29.6%) patients diagnosed with COPD, 61 (48.8%) patients with asthma, and 27 (21.6%) patients with COPD-A. The baseline study data are presented in Table 1. 

There were no significant differences observed in age among the groups. Also, the groups did not significantly differ in comorbidities profile. However, the COPD group exhibited a significantly higher proportion of male patients (*p* < 0.0001) and the lowest median of body mass index (BMI). Patients with COPD-A exhibited higher severity of dyspnea, measured using mMRC scale. The lowest mMRC scale at the end of RP presented patients with asthma. These patients also presented significantly lower final GINA-SCT evaluation results than patients with asthma-COPD coexistence. Differences in pulmonary function tests are typical for analyzed groups of patients (Table 1). 

Patients with asthma presented the lowest results in SGRQ, both in total score and in all domains (Table 1). 

The groups did not differ according to distance in 6 MWT (Table 1)

### 3.2. Assessment of the Effectiveness of the PR Program among Patients with Obstructive Lung Disease

Paired data analysis revealed significant improvement, both in CAT and GINA-SCT results (*p* < 0.00001 and *p* = 0.0001 respectively). Also, we observed significant improvement in mMRC dyspnoe scale (*p* < 0.00001), FEV_1_ (*p* < 0.00001), FVC (*p* < 0.00001) and distance in 6 MWT (*p* < 0.00001). We also observed significant improvement in SGRQ, both in total score (*p* < 0.00001) and each domain analysis (*p* < 0.00001). 

Analysing the differences in study parameters expressed as a relative difference post- vs. pre-PR, we did not observe significant differences among the study groups (Table 2), and therefore, post-hoc tests were not performed. 

### 3.3. Assessment of the Effectiveness of the PR Program among Patients with Asthma, COPD and COPD-A

#### 3.3.1. Exercise Tolerance Analysis

All groups of patients experienced a significant improvement in dyspnoea severity, measured by the mMRC scale (Table 3).

Also, among all three groups, we observed significant improvement in the distance measured during 6 MWT, with the highest improvement in the asthma group (Table 3).

#### 3.3.2. Spirometry Parameters

We observed improvement in both FEV_1_ and FVC in the asthma and COPD-A groups. We did not observe statistically significant differences in this context in the COPD group (Table 3). 

#### 3.3.3. Health-Related Quality of Life

SGRQ score improved in all three groups, in total score, as well as in all three domains (Table 3).

#### 3.3.4. Improvement of Exercise Tolerance Expressed as a Significant Elongation of the Distance in 6 MWT

The results of univariable and multivariable analysis are summarized in Table 4. 

**Figure 1:**
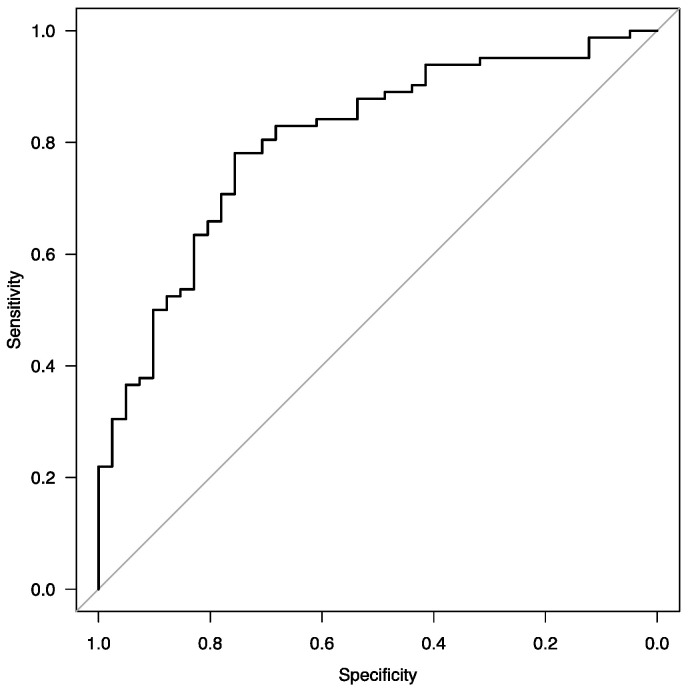
ROC curve of the final logistic regression model for assessing factors associated with significant improvement in 6 MWT.

The ROC curve of the final multivariable model is presented in Figure 1.

## 4. Discussion

The duration of a pulmonary rehabilitation (PR) program seems to be a pivotal determinant of its effectiveness. While consensus on the optimal duration remains elusive [17], studies indicate that longer programs tend to yield more favorable outcomes, regarding health-related quality of life in individuals with COPD [25]. Generally, an effective pulmonary rehabilitation program, inclusive of exercise training, is recommended to last at least six weeks [26]; however, this stipulation may pose constraints in various countries, due to financial limitations imposed by insurance companies and/or national health funds [1]. Therefore, our study aimed to assess the outcomes of an even shorter PR program across patients with COPD, asthma, and their coexistence, demonstrating significant improvements across various clinical parameters, including CAT, GINA-SCT, mMRC, FEV1, FVC, and 6 MWT results. These findings underscore the efficacy of the PR program in enhancing respiratory function and symptom control in the analyzed patient groups.

Of note, all patient groups experienced a significant reduction in dyspnea severity, as indicated by improvements in the mMRC scale. Additionally, improvements in 6 MWT distance were observed across all groups, with the greatest enhancement noted in the asthma group. This consistent improvement highlights the beneficial effects of PR on dyspnea management and exercise tolerance, regardless of the underlying respiratory condition.

However, while FEV_1_ and FVC improvements were evident in the asthma and COPD-A groups, statistical significance was not reached in the COPD group. This suggests a potential variation in the response to PR among analyzed populations, with asthma and COPD-A patients demonstrating more pronounced improvements in lung function, compared to COPD cases.

Significant enhancements were observed in the SGRQ scores across all patient groups, indicating a notable improvement in the quality of life following the PR intervention. These findings highlight the comprehensive benefits of PR beyond physiological parameters, encompassing aspects of daily functioning and well-being.

Furthermore, the multivariate logistic regression model identified several baseline parameters predictive of PR response, including primary 6 MWT distance, FVC, age, BMI, overweight status, and obesity. These factors collectively contribute to the likelihood of favorable outcomes following PR, providing valuable insights for patient selection and personalized intervention strategies.

The observed improvements in clinical outcomes, exercise tolerance, and quality of life reaffirm the effectiveness of PR as a therapeutic intervention for patients with analyzed conditions, even in such a short duration of the PR program as three weeks. Tailoring PR programs based on individual characteristics and addressing modifiable risk factors, such as overweight and obesity, may further optimize treatment outcomes. The findings underscore the importance of implementing PR, even in such reduced—three week–duration, as a cornerstone of comprehensive respiratory care, offering multifaceted benefits for patients with COPD, asthma and coexistence of these conditions.

Our results are concordant with the evidence supporting the efficacy of a three-week PR program within the COPD population. Notably, significant enhancements in parameters such as the 6 MWT, CAT, mMRC, and FEV_1_ were noted in severe COPD patients who underwent a three-week PR program [27]. In turn, von Leupoldt et al. reported notable improvements in exercise capacity, dyspnea, and Health-Related Quality of Life (HRQL) following an intensive 3-week outpatient PR regimen, irrespective of COPD severity [28]. Additionally, intriguing findings suggest that a three-week sanatorium rehabilitation program yielded augmented exercise capacity and improved self-assessment of health in patients with COPD and asthma. That program encompassed various modalities, such as breathing exercises, group exercises, inhalation therapy, magnetic therapy, crenotherapy, Sollux lamp irradiation, and massage. After such treatment, patients with COPD and asthma much better evaluated their health. Also, the distance covered in the 6 MWT increased by 9 m and 17.5 m in patients with COPD and asthma, respectively [29]. In the context of these observations, we achieved longer distances in 6 MWT; however we used very different rehabilitation programs, including endurance training. In the realm of asthma, Schultz and colleagues observed that individuals inadequately responsive to outpatient treatment may benefit from rehabilitation, with a 3-week PR course resulting in clinically relevant improvements in asthma control [30]. Similarly, Schneeberger et al. demonstrated significant enhancements in asthma control among patients undergoing a comprehensive three-week inpatient pulmonary rehabilitation program, particularly those with moderate to severe asthma [31]. 

However, our literature review did not identify any studies evaluating the effectiveness of a three-week program specifically in the COPD-A population. A search conducted by the authors revealed only one relevant study by Orooj et al., which assessed the efficacy of PR in COPD-A. This study observed favorable changes in functional capacity, health-related quality of life, and the BODE index following a six-week PR program in patients with concurrent asthma and COPD. However, the authors did not register changes in pulmonary function in these patients [32]. In contrast, our short three-week program yielded improvements in FEV1 and FVC, which was potentially attributable to differences in exercise program and training progression. Nonetheless, this phenomenon warrants further observation in a larger sample size.

Our study has several limitations, primarily its single-center, retrospective nature and low sample size, necessitating recognition as a hypothesis-generating study warranting replication in a more robust sample. The group size experienced a reduction, due to the impact of the COVID-19 pandemic. Additionally, the analyzed cohorts represent a significant but not exclusive subset of patients hospitalized in our department. Our rehabilitation services encompass a broad spectrum of pulmonary conditions, including interstitial lung diseases, scoliosis, and paralysis of the dome of the diaphragm. Notably, we serve as the sole PR center in our region. Another noteworthy aspect to consider is that exercise tolerance was exclusively evaluated using the 6 MWT, as opposed to the more expansive cardio-pulmonary exercise test (CPET), which will be implemented in our forthcoming prospective investigations. Finally, our study only focused on the short-term effect of PR. Nonetheless, we believe our study underscores the pivotal role of pulmonary rehabilitation in managing COPD, asthma, and their coexistence, while emphasizing the distinctiveness of, and the need to give special consideration to, the COPD-A group in the context of PR. 

## 5. Conclusions

The study provides compelling evidence supporting the short-term efficacy of the three-week PR in improving clinical outcomes, exercise tolerance, and quality of life in patients with COPD and asthma. Understanding the predictors of PR response and tailoring interventions accordingly can enhance treatment efficacy and optimize patient outcomes in analysed diseases. There is a need for further research, especially of the COPD-A group, to help develop a more individualized approach to this group of patients. Furthermore, further investigation is warranted of the sustainability of outcomes and the question of if a three-week PR program provides sufficient duration for the establishment of long-term adherence to health-enhancing behaviors.

## Figures and Tables

**Table 1:** Baseline data analysis. Abbreviations: BMI—body mass index, CAT—COPD Assessment Test, FEV_1_—forced expiratory volume in the first second, FVC—forced vital capacity, GINA-SCT—consensus-based GINA symptom control tool, IQR—interquartile range, mMRC—Modified Medical Research Council, PR—pulmonary rehabilitation, SD—standard deviation, SGRQ—St. George’s Respiratory Questionnaire, SpO_2_—peripheral capillary oxygen saturation, as measured by pulse oximetry, 6 MWT—six-minute walk test.

Parameter	COPD *N* = 37	Asthma *N* = 61	COPD-A *N* = 27	Total *N* = 125	*p*-Value
Age, years, median [IQR] Male sex, n (%) BMI, kg/m^2^, median [IQR]	71 [64–77] 24 (64.86) 24.7 [20.41–29.76]	69 [63–75] 13 (21.31) 27.9 [24.85–33.3]	70 [62–77] 11 (40.74) 27.52 [21.97–29.3]	70 [63–76] 48 (38.4) 26.33 [22.2–30.88]	0.58 **<0.0001** **0.02**
***Comorbidities*** Arterial hypertension Heart failure, n (%) History of MI, n (%) Diabetes mellitus type 2, n (%) Obesity, n (%) Cachexia, n (%) Chronic kidney disease, n (%) History of malignancy, n (%) History of stroke, n (%)	24 (64.86) 15 (40.54) 3 (8.11) 11 (29.73) 9 (24.32) 2 (5.41) 1 (2.7) 6 (16.22) 1 (2.7)	44 (72.13) 23 (37.7) 5 (8.2) 18 (29.51) 22 (36.07) 2 (3.28) 1 (1.63) 3 (4.92) 7 (11.48)	20 (74.07) 9 (33.33) 5 (18.52) 7 (25.93) 5 (18.52) 2 (7.4) 2 (7.4) 5 (18.52) 0 (0)	88 (70.4) 47 (37.6) 13 (10.4) 36 (28.8) 36 (28.8) 6 (4.8) 4 (3.2) 14 (11.2) 8 (6.4)	0.67 0.84 0.32 0.94 0.22 0.65 0.33 0.08 0.09
***Disease-specific scales*** CAT, points, mean (SD) GINA-SCT, points, median [IQR] mMRC, points, median [IQR]	22.07 (5.11) - 2 [2–3]	- 3 [1–3.25] 2 [1–3]	25.55 (6.47) 3 [3–4] 3 [2–3]	23.54 (5.87) 3 [2–4] 2 [2–2]	0.14 0.07 **0.04**
***6 MWT*** Distance, m, mean (SD) Δ SpO_2_, %, median [IQR]	330.14 (80.89) 0 [−1–2]	318.9 (84.31) 0 [−1–2]	311.11 (89.41) 0 [−1–1.5]	320.47 (84.07) 0 [−1–2]	0.66 0.86
***Spirometry*** FEV_1_, %, median [IQR] FVC, %, mean (SD) FEV1/FVC, median [IQR]	44 [33–52.5] 69.06 (13.89) 48.18 [44.24–53.82]	81 [65–94] 80.85 (22.04) 77.29 [70–81.59]	36 [24.5–48] 62.04 (19.71) 47.19 [40.73–54.56]	56 [39.75–81] 73.33 (20.87) 61.7 [48.16–77.27]	**<0.0001** **0.0001** **<0.0001**
***SGRQ*** total, points, mean (SD) impact, points, mean (SD) symptoms, points, mean (SD) activity, points median [IQR]	61.59 (14.28) 54.56 (20.02) 63.25 (15.18) 73.02 [59.46–79.67]	52.95 (17.23) 44.89 (20.89) 54.19 (19.44) 66.19 [59.46–79.67]	65.29 (15.43) 56.19 (20.87) 61.41 (14.28) 85.82 [78.98–92.51]	58.09 (16.77) 50.11 (21.13) 58.37 (17.65) 72.44 [59.46–85.87]	**0.002** **0.02** **0.03** **0.0001**

**Table 2:** Analysis of the differences in study parameters. Abbreviations: CAT—COPD Assessment Test, FEV_1_—forced expiratory volume in the first second, FVC—forced vital capacity, GINA-SCT—consensus-based GINA symptom control tool, mMRC—Modified Medical Research Council, SGRQ—St. George’s Respiratory Questionnaire, 6 MWT—six-minute walk test.

Parameter	Kruskal-Wallis Chi-Squared (*p*-Value)
***Disease-specific scales*** Δ CAT, points Δ GINA-SCT, points Δ mMRC, points	0.53 (*p* = 0.46) 0.18 (*p* = 0.67) 1.01 (*p* = 0.6)
***6 MWT*** Δ Distance, m	2.98 (*p* = 0.23)
***Spirometry*** Δ FEV_1_, % Δ FVC, %	4.68 (*p* = 0.1) 3.97 (*p* = 0.14)
***SGRQ*** Δ total, points Δ impact, points Δ symptoms, points Δ activity, points	1.34 (*p* = 0.51) 0.57 (*p* = 0.75) 5.13 (*p* = 0.08) 1.73 (*p* = 0.42)

**Table 3:** Analysis of paired data. Data were presented as the mean (SD), median [IQR] or n (%), depending on the character and distribution of data. Statistical significance: * < 0.05, ** < 0.01, *** < 0.0001, **** < 0.00001. Abbreviations: FEV_1_—forced expiratory volume in the first second, FVC—forced vital capacity, IQR—interquartile range, mMRC—Modified Medical Research Council, SD—standard deviation, SGRQ—St. George’s Respiratory Questionnaire, SpO_2_—peripheral capillary oxygen saturation as measured by pulse oximetry, 6 MWT—six-minute walk test.

Parameter	COPD		Asthma		COPD-A	
	Pre-PR	Post-PR	Pre-PR	Post-PR	Pre-PR	Post-PR
***Disease-specific scales*** CAT, points, mean (SD) GINA-SCT, points, median [IQR] mMRC, points, median [IQR]	22.07 (5.11) - 2 [2–3]	16.93 *** (5.6) - 1 **** [1–2]	- 3 [1–3.25] 2 [1–3]	- 0.5 ** [0–1.25] 1 **** [0–1]	25.55 (6.47) 3 [3–4] 3 [2–3]	18.91 ** **(7.94)** 2 * **[1–3.75]** 1 ** **[0.5–2]**
***6 MWT*** Distance, m, mean (SD) Δ SpO_2_, %, median [IQR]	330.14 (80.89) 0 [–1–2]	388.58 **** (74.92) 1 [−0.25–3]	318.9 (84.31) 0 [–1–2]	403.84 **** (98.65) 1 [0–2]	311.11 (89.41) 0 [−1–1.5]	378.41 **** (95.12) 1 [−0.5–2.5]
***Spirometry*** FEV_1_, %, median [IQR] FVC, %, mean (SD) FEV1/FVC, median [IQR]	44 [33–52.5] 69.06 (13.89) 48.18 [44.24–53.82]	43 [35–56] 71.46 (16.86) 48.57 [42.01–54.49]	81 [65–94] 80.85 (22.04) 77.29 [70–81.59]	86 ** [73–98] 87.49 *** (20.65) 76.85 [71.02–81.88]	36 [24.5–48] 62.04 (19.71) 47.19 [40.73–54.56]	43 ** [33.5–57.5] 70.52 ** (20.17) 46.23 [42.46–57.41]
***SGRQ*** total, points, mean (SD) impact, points, median [IQR] symptoms, points, median [IQR] activity, points, median [IQR]	61.59 (14.28) 55.38 [41.41–69.16] 63.12 [53.12–76.01] 73.02 [59.46–79.67]	49.63 **** (18.11) 39.6 *** [23.04–58.65] 42.24 *** [24.56–59.9] 66.1 * [48.05–79.27]	52.95 (17.23) 44.36 [28.12–61.49] 52.64 [40.71–68.94] 66.19 [59.46–79.67]	37.97 **** **(21.96)** 25.83 *** **[11.98–52.35]** 34.1 **** **[16.76–53.87]** 59.3 **** **[35.29–66.31]**	65.29 (15.43) 52.18 [44.01–72.13] 60.56 [54.89–72.69] 85.82 [78.98–92.51]	50.51 *** **(21.13)** 42.5 ** **[23.95–57.78]** 43.86 ** **[29.19–65.46]** 72.76 ** **[54.74–85.54]**

**Table 4:** Univariable and multivariable analysis results. Data were presented as odds ratio and 96%CIs. Abbreviations: BMI—body mass index, CAT—COPD Assessment Test, FEV_1_—forced expiratory volume in the first second, FVC—forced vital capacity, GINA-SCT—consensus-based GINA symptom control tool, IQR—interquartile range, mMRC—Modified Medical Research Council, PR—pulmonary rehabilitation, SD—standard deviation, SGRQ—St. George’s Respiratory Questionnaire, SpO_2_—peripheral capillary oxygen saturation, as measured by pulse oximetry, 6 MWT—six-minute walk test.

Parameter	Odds Ratio (95%CI)	*p*-Value
***Univariable*** Age Sex BMI Overweight Obesity Arterial hypertension Heart failure History of MI Diabetes mellitus type 2 Chronic kidney disease History of malignancy History of stroke CAT GINA-SCT mMRC primary distance in 6 MWT Δ SpO_2_ in 6 MWT %FEV_1_ %FVC FEV1/FVC SRGQ—total SRGQ—impact SRGQ—symptoms SRGQ—activity	0.96 (0.92–1.01) 0.65 (0.31–1.39) 0.93 (0.88–0.99) 2.54 (1.08–6.00) 0.44 (0.2–0.97) 0.54 (0.23–1.27) 0.53 (0.25–1.13) 0.79 (0.24–2.58) 0.72 (0.32–1.61) 0.49 (0.06–3.64) 0.62 (0.2–1.93) 0.83 (0.19–3.67) 1.02 (0.88–1.18) 1.08 (0.62–1.86) 0.85 (0.55–1.32) 1.0 (0.99–1.0) 1.03 (0.9–1.17) 1.01 (1.0–1.03) 1.02 (1.0–1.04) 1.01 (0.98–1.03) 0.99 (0.97–1.01) 0.99 (0.97–1.01) 0.99 (0.97–1.01) 1.01 (0.99–1.02)	0.08 0.27 0.03 0.03 0.04 0.16 0.1 0.7 0.43 0.49 0.41 0.81 0.81 0.79 0.5 0.18 0.7 0.12 0.03 0.58 0.32 0.22 0.26 0.59
***Multivariable*** primary distance in 6 MWT %FVC Age BMI Overweight Obesity	0.99 (0.98–0.997) 1.04 (1.01–1.06) 0.92 (0.86–0.97) 0.78 (0.65–0.94) 10.6 (2.18–51.5) 14 (0.96–203)	0.004 0.004 0.004 0.008 0.003 0.05

## Data Availability

The data presented in this study are available from the corresponding author upon reasonable request.
